# Healthcare resource utilization and costs associated with psychiatric comorbidities in pediatric patients with attention-deficit/hyperactivity disorder: a claims-based case-cohort study

**DOI:** 10.1186/s13034-024-00770-8

**Published:** 2024-07-08

**Authors:** Jeff Schein, Martin Cloutier, Marjolaine Gauthier-Loiselle, Rebecca Bungay, Kathleen Chen, Deborah Chan, Annie Guerin, Ann Childress

**Affiliations:** 1 Otsuka Pharmaceutical Development and Commercialization, Inc., 508 Carnegie Center, Princeton, NJ 08540 USA; 2 Analysis Group, Inc., 1190 avenue des Canadiens-de-Montréal, Tour Deloitte, Suite 1500, Montréal, QC H3B 0G7 Canada; 3 Center for Psychiatry and Behavioral Medicine, 7351 Prairie Falcon Rd STE 160, Las Vegas, NV 89128 USA

**Keywords:** Attention deficit hyperactivity disorder, Psychiatric disorders, Anxiety, Depression, Comorbidity, Healthcare resource use, Costs, Child, Adolescent

## Abstract

**Background:**

Attention-deficit/hyperactivity disorder (ADHD) has been shown to pose considerable clinical and economic burden; however, research quantifying the excess burden attributable to common psychiatric comorbidities of ADHD among pediatric patients is scarce. This study assessed the impact of anxiety and depression on healthcare resource utilization (HRU) and healthcare costs in pediatric patients with ADHD in the United States.

**Methods:**

Patients with ADHD aged 6–17 years were identified in the IQVIA PharMetrics Plus database (10/01/2015-09/30/2021). The index date was the date of initiation of a randomly selected ADHD treatment. Patients with ≥ 1 diagnosis for anxiety and/or depression during both the baseline (6 months pre-index) and study period (12 months post-index) were classified in the ADHD+anxiety/depression cohort; those without diagnoses for anxiety nor depression during both periods were classified in the ADHD-only cohort. Entropy balancing was used to create reweighted cohorts. All-cause HRU and healthcare costs during the study period were compared using regression analyses. Cost analyses were also performed in subgroups by comorbid conditions.

**Results:**

The reweighted ADHD-only cohort (*N* = 204,723) and ADHD+anxiety/depression cohort (*N* = 66,231) had similar characteristics (mean age: 11.9 years; 72.8% male; 56.2% had combined inattentive and hyperactive ADHD type). The ADHD+anxiety/depression cohort had higher HRU than the ADHD-only cohort (incidence rate ratios for inpatient admissions: 10.3; emergency room visits: 1.6; outpatient visits: 2.3; specialist visits: 5.3; and psychotherapy visits: 6.1; all *p* < 0.001). The higher HRU translated to greater all-cause healthcare costs; the mean per-patient-per-year (PPPY) costs in the ADHD-only cohort vs. ADHD+anxiety/depression cohort was $3,988 vs. $8,682 (*p* < 0.001). All-cause healthcare costs were highest when both comorbidities were present; among patients with ADHD who had only anxiety, only depression, and both anxiety and depression, the mean all-cause healthcare costs were $7,309, $9,901, and $13,785 PPPY, respectively (all *p* < 0.001).

**Conclusions:**

Comorbid anxiety and depression was associated with significantly increased risk of HRU and higher healthcare costs among pediatric patients with ADHD; the presence of both comorbid conditions resulted in 3.5 times higher costs relative to ADHD alone. These findings underscore the need to co-manage ADHD and psychiatric comorbidities to help mitigate the substantial burden borne by patients and the healthcare system.

**Supplementary Information:**

The online version contains supplementary material available at 10.1186/s13034-024-00770-8.

## Introduction

Attention-deficit/hyperactivity disorder (ADHD) is one of the most common neurodevelopmental disorders of childhood. According to national surveys in the United States (US), the estimated 12-month prevalence of ADHD was 10.0% among children and 6.5% among adolescents [1–3], although estimates in the literature range from 3.4 to 11.1% for children and 3.4–13.6% for adolescents [3–8]. Pediatric patients with ADHD are at increased risk of numerous behavioral and psychophysiological problems relative to their non-ADHD counterparts [9–11], and the condition is estimated to add $33.2 billion of excess costs to the US society each year [12]. 

ADHD is a heterogeneous disorder partially exemplified by its extensive psychiatric comorbidities, among which anxiety and depression are common [3, 13]. The presence of psychiatric comorbidities among pediatric patients with ADHD has been shown to exacerbate patient functioning in social and educational domains relative to ADHD alone [14–16]. Furthermore, psychiatric comorbidities may confound the management of ADHD by increasing the need for care coordination, use of medications, and time to reach optimal symptom management [17, 18]. Patients with comorbid anxiety and depression in ADHD have also been associated with more treatment changes and increased healthcare costs compared to those with ADHD alone [19]. 

Although ample evidence has demonstrated that pediatric patients with ADHD have increased healthcare resource utilization (HRU) and expenditures compared to those without ADHD [20–22], data on the excess burden imposed by common psychiatric comorbidities are relatively limited. Such knowledge may help inform stakeholders, including patients, parents, teachers, clinicians, and the healthcare system, on tailored strategies for managing ADHD in pediatric patients. The current study aimed to partially address this research gap by quantifying the impact of comorbid anxiety and/or depression on HRU and healthcare costs in pediatric patients with ADHD in the US using a large claims database.

## Methods

### Data source

Claims data from the IQVIA PharMetrics® Plus (IQVIA) database (10/01/2015-09/30/2021) were used. The database comprises over 190 million unique beneficiaries with a diverse representation of geographic areas, employers, providers, and payers across the US. Information includes inpatient and outpatient diagnoses and procedures, prescription fills, patients’ pharmacy and medical benefits, inpatient stays, and provider details. Additional data elements encompass dates of service, demographic variables, plan type, payer type, and start and stop dates of health plan enrollment. Economic variables include the actual amount paid by health plans to the provider for services rendered. Data analyzed in this study are de-identified and comply with the patient requirements of the Health Insurance Portability and Accountability Act (HIPAA); therefore, no review by an institutional review board nor informed consent was required per Title 45 of CFR, Part 46.101(b)(4) [23]. 

### Study design

This study used a retrospective case-cohort design [24]. The index date was defined as the date of initiation of a randomly selected US Food and Drug Administration (FDA)–approved agent for the treatment of ADHD that was newly initiated after the first ADHD diagnosis; this definition allowed capturing of patients at different stages of their ADHD treatment journeys. The baseline period was defined as the 6-month period prior to the index date. The study period was defined as the 12-month period following the index date.

### Selection criteria and patient populations

Patients were included in the study if they met the following inclusion criteria: (1) had ≥ 2 ADHD diagnoses (International Classification of Diseases, Tenth Revision, Clinical Modification [ICD-10-CM] F90.x) recorded on distinct dates; (2) had ≥ 1 prescription fill for an FDA-approved pharmacological treatment for ADHD on or after the first ADHD diagnosis; (3) had continuous health plan enrollment, including both medical and pharmacy coverage, during the baseline and study periods; and (4) were aged ≥ 6 years at the start of the baseline period and ≤ 17 years at the end of the study period (Supplementary Figure**S1**).

Eligible patients were classified into two study cohorts: patients with ≥ 1 diagnosis for anxiety and/or depression (as defined by the Diagnostic and Statistical Manual of Mental Disorders, Fifth Edition [DSM-5] [4]) recorded on a medical claim during both the baseline and study periods were classified into the *ADHD+anxiety/depression cohort*, and those without diagnoses for anxiety nor depression recorded on a medical claim during the baseline and study periods were classified into the *ADHD-only cohort*.

For cost analyses, patients in the ADHD+anxiety/depression cohort were further stratified into three subgroups by comorbid conditions: patients with ADHD who, during both the baseline and study periods, only had comorbid anxiety were classified as the *ADHD+only anxiety subgroup*, those who only had comorbid depression were classified as the *ADHD+only depression subgroup*, and those with both anxiety and depression were classified as the *ADHD+both anxiety and depression subgroup*.

### Outcome measures

Baseline demographic, clinical, and treatment characteristics were measured based on information recorded in claims data. In particular, psychiatric and physical comorbidities were identified based on ICD-10-CM diagnosis codes recorded on medical claims, and treatments were identified based on pharmacy claims for prescription fills. All-cause HRU and healthcare costs during the study period were assessed. All-cause HRU was reported per-patient-per-year (PPPY) by categories (i.e., inpatient admissions, days with emergency room visits, days with outpatient visits, number of specialist visits [i.e., psychiatrist, neurologist], and psychotherapy visits). All-cause healthcare costs were reported PPPY and included pharmacy and medical costs; the latter comprised of costs related to inpatient, emergency room, and outpatient visits. Costs were assessed from the payer’s perspective (i.e., health plan payment + coordination of benefits, excluding patients’ payment) and adjusted to 2022 US dollars using the medical component of the Consumer Price Index.

### Statistical analysis

Entropy balancing was used to create reweighted cohorts [25], with the following baseline characteristics considered: age, sex, region, health plan type, type of ADHD, calendar year of index date, number of agents received on or after ADHD diagnosis and before the index date, time from first observed ADHD diagnosis to first ADHD treatment, and time from first observed diagnosis to index date. Comorbidities related to anxiety and depression were excluded from the entropy balancing as these comorbidities may be a part of the disease burden assessed in this study. Baseline characteristics of the balanced cohorts were summarized using descriptive statistics, with means, medians, and standard deviations reported for continuous variables, and frequency counts and percentages reported for categorical variables. Standardized differences between the cohorts were calculated, with values < 0.10 being considered as well balanced [26]. 

All-cause HRU was compared between balanced cohorts using weighted negative binomial regression models with a log link and reported as incidence rate ratios along with their 95% confidence intervals and p-values.

All-cause healthcare costs were compared between the balanced cohorts using weighted two-part regression models. In addition, cost analyses were also performed between the ADHD-only cohort and each of the comorbidity subgroups (i.e., ADHD+only anxiety, ADHD+only depression, and ADHD+both anxiety and depression) to assess the excess cost burden attributable to the respective comorbid conditions.

All analyses were performed using SAS Enterprise guide 7.1 and Stata 16.

## Results

### Sample size

A total of 270,954 patients met the cohort-specific inclusion criteria, including 204,723 patients in the ADHD-only cohort and 66,231 patients in the ADHD+anxiety/depression cohort. Stratified by comorbid conditions, 41,992 patients were included in the ADHD+only anxiety subgroup, 14,054 in the ADHD+only depression subgroup, and 10,185 in the ADHD+both anxiety and depression subgroup (Supplementary Figure**S1**).

### Baseline patient characteristics

Demographic and clinical characteristics were well balanced between the reweighted cohorts (Table 1). Mean patient age was 11.9 years, and the majority were male (72.8%) and had combined inattentive and hyperactive ADHD type (56.2%). The proportion of patients with baseline psychiatric and physical comorbidities were overall higher in the ADHD+anxiety/depression cohort than the ADHD-only cohort. Apart from anxiety and depression, the most prevalent psychiatric comorbidities with a standardized difference of > 0.10 between the cohorts included neurodevelopmental disorders other than ADHD (ADHD-only vs. ADHD+anxiety/depression: 7.8% vs. 20.7%), trauma- and stressor-related disorders (5.7% vs. 15.4%), and disruptive, impulse-control, and conduct disorders (4.7% vs. 15.9%). The most prevalent physical comorbidity with a standardized difference > 0.10 between the cohorts was cardiac arrhythmias (ADHD-only vs. ADHD+anxiety/depression: 1.8% vs. 3.7%).

**Table 1:** Patient demographic, clinical, and treatment characteristics of weighted cohorts

	ADHD-only cohort *N* = 204,723	ADHD+anxiety/depression cohort^a^ *N* = 66,231	Standardized difference^b^
Demographic characteristics as of index date			
**Age (years)**, mean ± SD [median]	11.9 ± 2.9 [11.8]	11.9 ± 2.9 [11.8]	0.00
**Sex**, n (%)			
Male	149,077 (72.8%)	48,229 (72.8%)	0.00
Female	55,646 (27.2%)	18,002 (27.2%)	0.00
**Region**^**c**^, n (%)			
South	99,717 (48.7%)	32,260 (48.7%)	0.00
Midwest	56,583 (27.6%)	18,305 (27.6%)	0.00
Northeast	31,258 (15.3%)	10,112 (15.3%)	0.00
West	17,038 (8.3%)	5,512 (8.3%)	0.00
**Health plan type**^**c**^, n (%)			
Preferred provider organization	161,833 (79.0%)	52,356 (79.0%)	0.00
Health maintenance organization	22,338 (10.9%)	7,227 (10.9%)	0.00
Point of service	13,603 (6.6%)	4,401 (6.6%)	0.00
Consumer directed health care	4,924 (2.4%)	1,593 (2.4%)	0.00
Indemnity/traditional	1,992 (1.0%)	644 (1.0%)	0.00
**Clinical characteristics during baseline**			
**Time from first observed ADHD diagnosis to first ADHD treatment (months)**, mean ± SD [median]	**1.4 ± 4.4 [0.1]**	**1.4 ± 4.4 [0.1]**	**0.00**
**Time from first observed ADHD diagnosis to index date (months)**, mean ± SD [median]	**13.8 ± 12.7 [10.2]**	**13.8 ± 12.7 [9.8]**	**0.00**
**Number of ADHD treatments received between diagnosis and index date**, mean ± SD [median]	**2.3 ± 2.5 [2.0]**	**2.3 ± 2.5 [2.0]**	**0.00**
0, n (%)	43,735 (21.4%)	14,149 (21.4%)	0.00
1, n (%)	53,153 (26.0%)	17,196 (26.0%)	0.00
≥ 2, n (%)	107,835 (52.7%)	34,886 (52.7%)	0.00
**Type of ADHD at most recent diagnosis on or prior to index date**, n (%)			
Combined presentation	114,954 (56.2%)	37,189 (56.2%)	0.00
Inattentive	50,233 (24.5%)	16,251 (24.5%)	0.00
Hyperactive	12,075 (5.9%)	3,906 (5.9%)	0.00
Other/unspecified	27,461 (13.4%)	8,884 (13.4%)	0.00
**Psychiatric comorbidities**^**d**^, n (%)			
Anxiety	0 (0.0%)	53,784 (81.2%)	2.94^†^
Depression	0 (0.0%)	26,453 (39.9%)	1.15^†^
Neurodevelopmental disorders (excluding ADHD)	15,917 (7.8%)	13,712 (20.7%)	0.38^†^
Trauma- and stressor-related disorders	11,742 (5.7%)	10,213 (15.4%)	0.32^†^
Disruptive, impulse-control, and conduct disorders	9,686 (4.7%)	10,526 (15.9%)	0.37^†^
Sleep-wake disorders	5,220 (2.5%)	5,219 (7.9%)	0.24^†^
Elimination disorders	1,632 (0.8%)	1,179 (1.8%)	0.09
Obsessive-compulsive and related disorders	1,141 (0.6%)	2,831 (4.3%)	0.24^†^
Bipolar and related disorders	618 (0.3%)	6,193 (9.4%)	0.43^†^
**Most common physical comorbidities**^**e**^, n (%)			
Chronic pulmonary disease	12,284 (6.0%)	5,422 (8.2%)	0.09
Other neurological disorders^f^	3,538 (1.7%)	2,060 (3.1%)	0.09
Obesity	3,352 (1.6%)	1,855 (2.8%)	0.08
Cardiac arrhythmias	1,539 (0.8%)	1,244 (1.9%)	0.10^†^
**Treatment characteristics**			
**ADHD treatment initiated at index date**^**g**^, n (%)			
Stimulant	187,029 (91.4%)	52,650 (79.5%)	0.34†
Non-stimulants	20,938 (10.2%)	15,055 (22.7%)	0.34†
**Pharmacological treatments during baseline**^**g**^, n (%)			
**ADHD treatments**	**155,844 (76.1%)**	**49,778 (75.2%)**	**0.02**
Stimulants	147,380 (72.0%)	42,745 (64.5%)	0.16†
Non-stimulants	21,461 (10.5%)	13,964 (21.1%)	0.29†
**Antianxiety agents**	**2,543 (1.2%)**	**5,050 (7.6%)**	**0.31**†
**Antidepressants**	**9,494 (4.6%)**	**33,783 (51.0%)**	**1.21**†
**Non-pharmacological treatments during baseline** ^**h**^			
**Psychotherapy**, n (%)	**30,852 (15.1%)**	**40,119 (60.6%)**	**1.06**†
Number of psychotherapy visits, mean ± SD [median]	5.0 ± 5.3 [3.0]	7.4 ± 6.9 [5.0]	0.39**†**
**Specialist visits**, n (%)	**24,968 (12.2%)**	**29,087 (43.9%)**	**0.76†**
Number of specialist visits, mean ± SD [median]	2.5 ± 4.0 [2.0]	3.8 ± 4.8 [2.0]	0.29†

ADHD, attention-deficit/hyperactivity disorder; SD, standard deviation

^†^Indicates standardized differences > 0.10

**Notes**:

^a^The ADHD+anxiety/depression cohort comprised patients with ADHD+only anxiety, ADHD+only depression, and ADHD+both anxiety and depression

^b^A standardized difference of < 0.10 was considered as well balanced

^c^In both cohorts, ≤ 0.1% reported “Unknown”

^d^Based on American Psychiatric Pub (2022). Diagnostic and Statistical Manual of Mental Disorders (DSM-5-TR®)

^e^Based on Elixhauser A, Steiner C, Kruzikas. D. HCUP Comorbidity Software. Healthcare Cost and Utilization Project (HCUP). October 2015. Agency for Healthcare Research and Quality, Rockville, MD. Available from: https://www.hcup-us.ahrq.gov/toolssoftware/comorbidity/comorbidity.jsp#download

^f^Includes the following conditions: gangliosidosis/sphingolipidosis, systemic atrophies primarily affecting the central nervous system (i.e., Huntington’s), extrapyramidal and movement disorders (i.e., Parkinson’s), other degenerative diseases of the nervous system (i.e., Alzheimer’s), demyelinating diseases of the central nervous system (i.e., multiple sclerosis), episodic and paroxysmal disorders (i.e., epilepsy and seizures), cerebral palsy, and hydrocephalus

^g^Categories are not mutually exclusive

^h^Calculated among those with ≥ 1 visit

Although the majority of patients received stimulants as the index treatment in both cohorts, the proportion of patients who received non-stimulants was observed to be numerically higher in the ADHD+anxiety/depression cohort (22.7%) than in the ADHD-only cohort (10.2%); similar trends were observed during the baseline period (Table 1). Furthermore, more patients in the ADHD+anxiety/depression cohort than the ADHD-only cohort received antianxiety agents or antidepressants, or had psychotherapy or specialist visits, during the baseline period.

### HRU

**Fig. 1:**
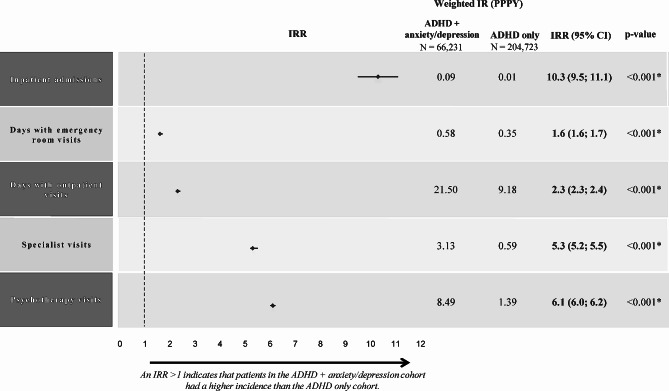
All-cause HRU during the 12-month study period^a^. ADHD, attention-deficit/hyperactivity disorder; CI, confidence interval; IR, incidence rate; IRR, incidence rate ratio; PPPY, per-patient-per-year. *Statistically significant at the 0.1% level. ^a^The ADHD+anxiety/depression cohort comprised patients with ADHD+only anxiety, ADHD+only depression, and ADHD+both anxiety and depression

During the study period, rates of all-cause HRU were significantly higher in the ADHD+anxiety/depression cohort than in the ADHD-only cohort, with an incidence rate ratio of 10.3 for inpatient admissions, 1.6 for emergency room visits, 2.3 for outpatient visits, 5.3 for specialist visits, and 6.1 for psychotherapy visits (all *p* < 0.001; Fig. 1).

### Healthcare costs

**Fig. 2:**
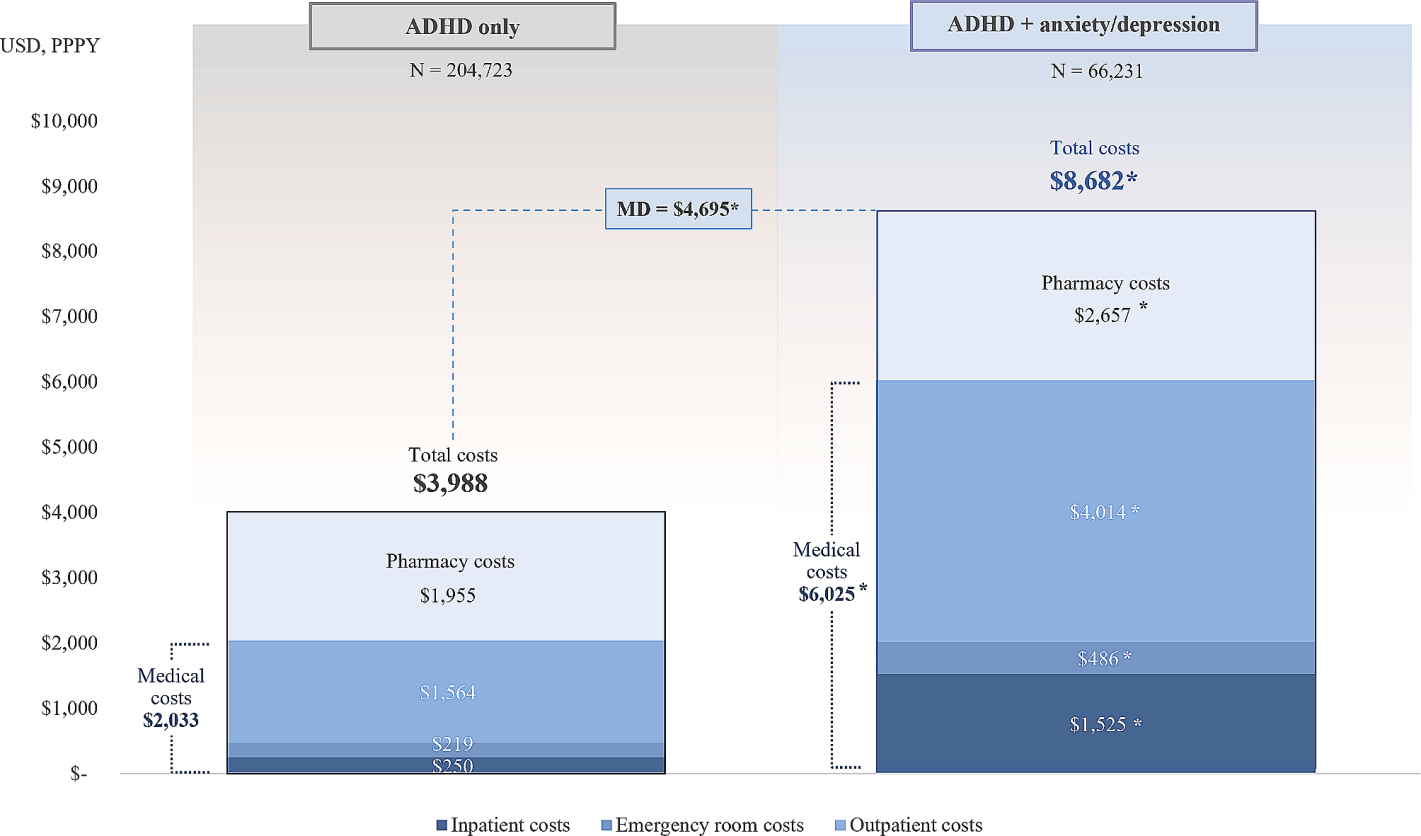
All-cause healthcare costs during the 12-month study period^a^. ADHD, attention-deficit/hyperactivity disorder; MD, mean difference; PPPY, per-patient-per-year; USD, United States dollar. *Statistically significantly different than the ADHD-only cohort at the 0.1% level. ^a^The ADHD+anxiety/depression cohort comprised patients with ADHD+only anxiety, ADHD+only depression, and ADHD+both anxiety and depression

Compared with the ADHD-only cohort, the mean all-cause healthcare costs PPPY were more than twice as high in the ADHD+anxiety/depression cohort ($3,988 vs. $8,682; mean difference: $4,695), driven by differences in both medical and pharmacy costs (all *p* < 0.001; Fig. 2).

**Fig. 3:**
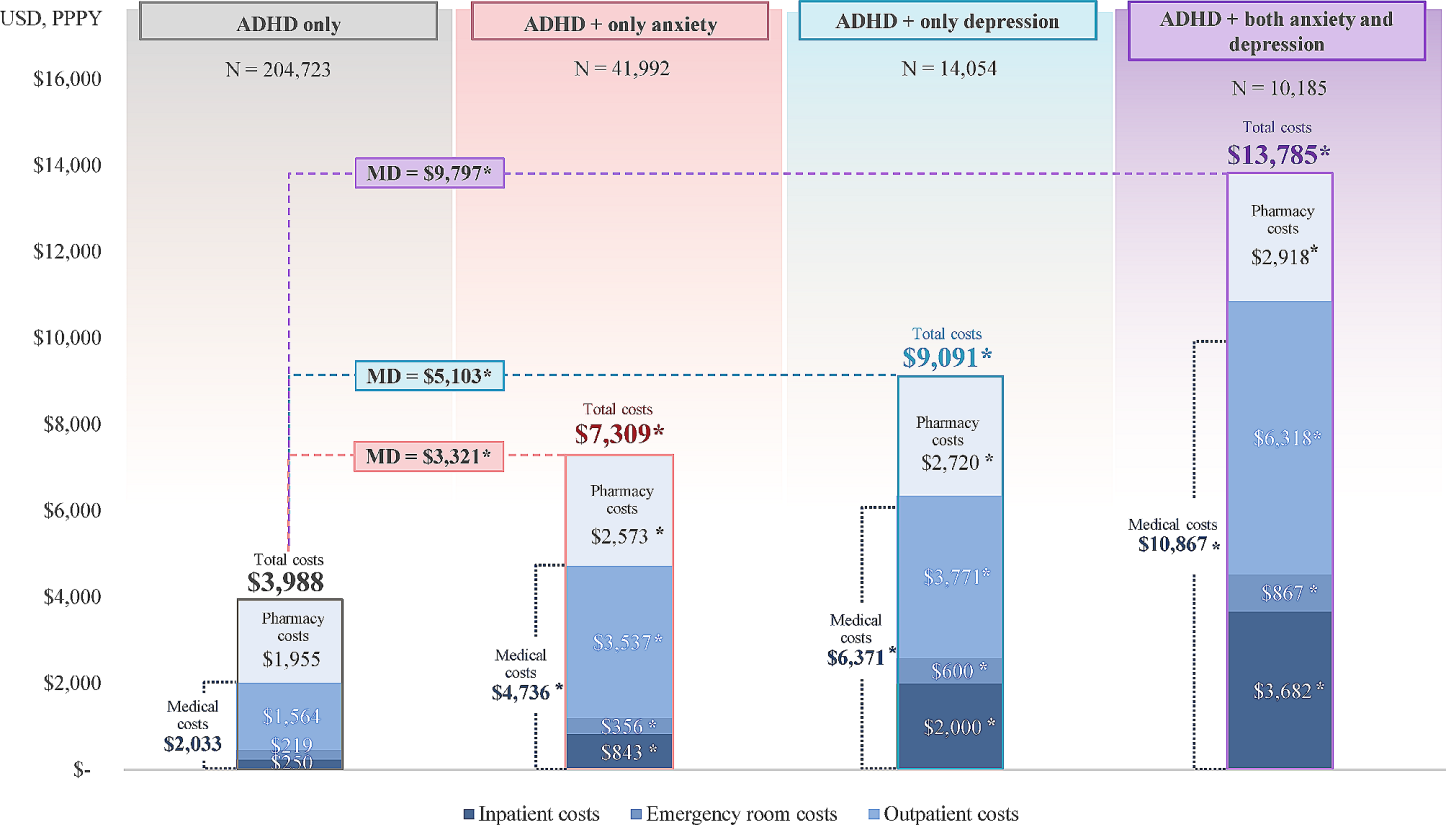
All-cause healthcare costs during the 12-month study period– subgroup analysis. ADHD, attention-deficit/hyperactivity disorder; MD, mean difference; PPPY, per-patient-per-year; USD, United States dollar. *Statistically significantly different than the ADHD-only cohort at the 0.1% level

Among the subgroups of patients with ADHD, the mean all-cause healthcare costs PPPY were $7,309 among those who had only anxiety, $9,901 among those who had only depression, and $13,785 among those who had both anxiety and depression (all *p* < 0.001; Fig. 3), translating to 1.8, 2.5, and 3.5 times higher excess healthcare costs compared with those who had ADHD only.

## Discussion

**Fig. 4:**
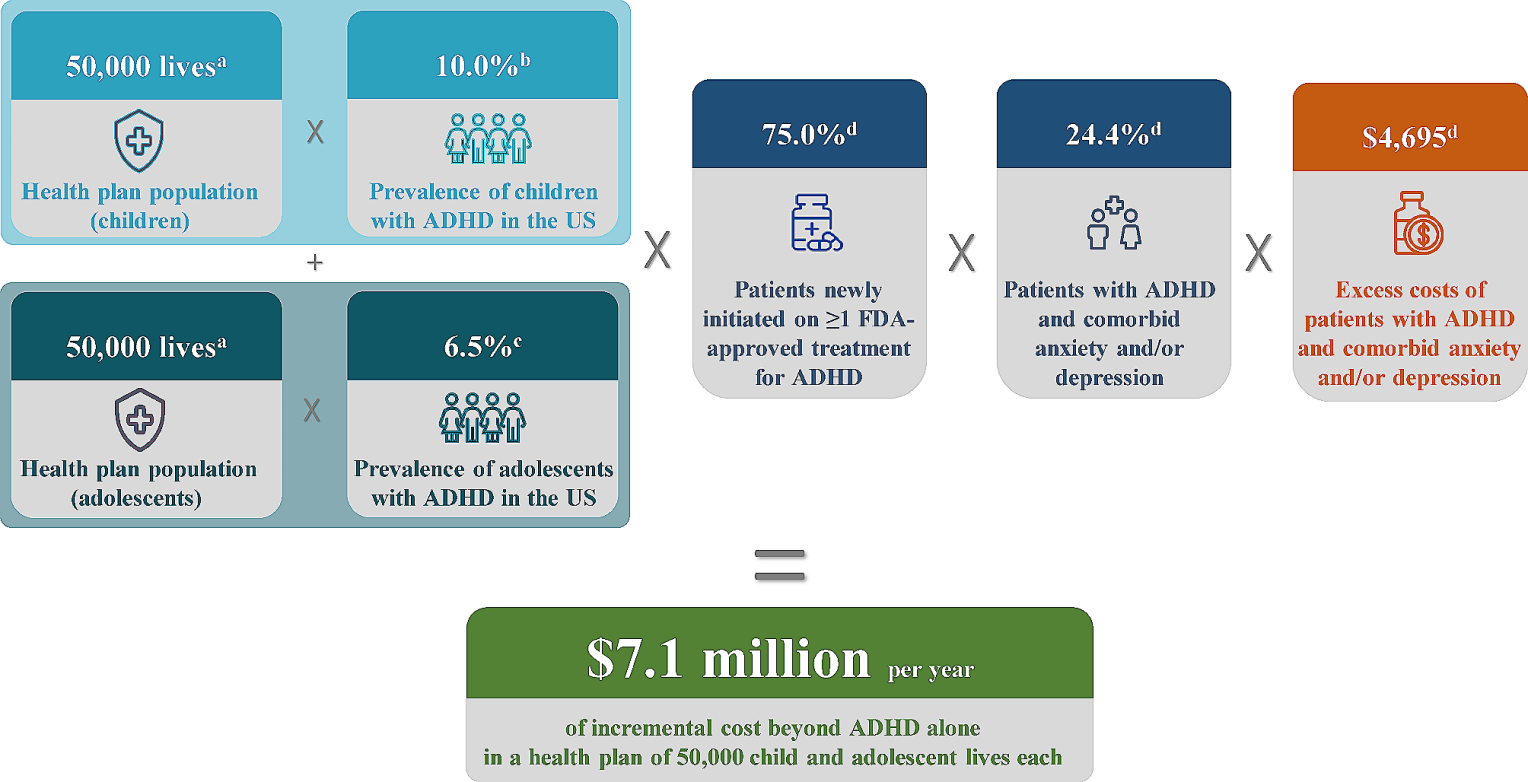
Incremental cost of comorbid anxiety and/or depression in ADHD relative to ADHD alone in a simulated health plan. ADHD, attention-deficit/hyperactivity disorder; FDA, Food and Drug Administration; US, United States. ^a^A hypothetical value. ^b^Based on the National Survey of Children’s Health data, 2018 [ 2 ]. ^c^Based on Kessler et al., 2012 [ 3 ]. ^d^Based on results in the current study

The current case-cohort analysis found that relative to pediatric patients with ADHD only, those with ADHD and comorbid anxiety and/or depression were 10 times more likely to be hospitalized and 5–6 times more likely to have a specialist or psychotherapy visit. Furthermore, pediatric patients with ADHD and comorbid anxiety and/or depression incurred more than twice the healthcare costs of those without the comorbidities, with a mean difference of $4,695 PPPY. Notably, the excess healthcare cost further increased among those with both comorbid anxiety and depression, with the total cost being 3.5 times higher than those with ADHD only. Together, these findings underscore the substantial excess HRU and cost burden imposed by comorbid anxiety and depression among pediatric patients with ADHD. The excess per-patient costs associated with these comorbidities could amount to an enormous expenditure for payers. For example, in a hypothetical health plan of 100,000 lives (with 50,000 children and adolescents, respectively), the excess healthcare cost of $4,695 PPPY could amount to $7.1 million per year incremental to the cost burden associated with ADHD alone (Fig. 4). Sensitivity analyses varying ADHD prevalence estimates suggest that the incremental healthcare cost of comorbid anxiety and depression relative to ADHD alone in a hypothetical health plan of 100,000 lives could range from $2.9 to $10.6 million per year assuming an ADHD prevalence of 3.4–11.1% for children and 3.4–13.6% for adolescents [3–8]. 

Findings of the current study are generally similar to a previous study among adults with ADHD, which found that comorbid anxiety and/or depression, especially when presented together, contributed to significant excess HRU and healthcare costs [27]. It has been previously shown that patients with ADHD who have comorbid anxiety and/or depression exhibit higher odds of treatment changes and incur higher associated costs relative to those with ADHD only [19], which may have partially contributed to the poorer clinical and economic outcomes among those with the comorbidities observed in the current study. It was also noted that treatment characteristics appear to be different among the pediatric and adult populations: a numerically higher proportion of pediatric patients received non-stimulants when anxiety and/or depression were present relative to adults with these comorbidities (22.7% vs. 6.8% at index; 21.1% vs. 5.3% during baseline) [27]. This may be due to some physicians being more cautious when considering stimulants for at-risk pediatric patients with comorbid emotional or behavioral conditions given the potential side effects of some medications on mood and emotion symptoms, despite their effectiveness [1]. Collectively, these observations highlight the need for improved management and treatment options to alleviate the burden associated with comorbidities in ADHD [28]. 

The current findings are also in line with the literature on HRU and economic burden of comorbidities among pediatric patients with ADHD [18, 29, 30]. A prior large US national survey reported that a higher proportion of pediatric patients with ADHD and comorbid emotional disturbances and depression, respectively, were hospitalized compared with those with the comorbidity only, and inpatient costs tended to be higher with the presence of both ADHD and the comorbidity [30]. Another US national survey found significantly greater odds of HRU (i.e., having ≥ 6 healthcare provider visits; ≥2 emergency room visits) among pediatric patients with ADHD and comorbid anxiety and depression, respectively [29]. Relative to the previous studies, the current study comprises more comprehensive HRU and cost categories, which provides insight on the overall clinical and economic burden attributable to anxiety and depression among those with ADHD. Furthermore, cost analyses among comorbidity subgroups also quantify the substantial impact to the healthcare system when both comorbid anxiety and depression are present in ADHD. Future studies investigating the impact of other psychiatric comorbidities on outcomes of pediatric patients with ADHD are warranted.

Managing ADHD and psychiatric comorbidities early in pediatric patients with ADHD may mitigate their burden in both the short-term and longer-term. In the short-term, reduced hospitalizations and psychotherapy visits may lead to less missed school days and better academic performances [31]. In the longer-term, certain childhood comorbidities, including anxiety and depression, have been shown to predict the development of other psychiatric comorbidities in later life [32]; therefore, a larger ADHD population may be affected by the impact of psychiatric comorbidities if the ADHD and its comorbid symptoms are not managed early. Indeed, the prevalence of comorbid anxiety and depression in ADHD appears to increase with age from childhood to adulthood [18, 19, 33]. In US population-based surveys, comorbid anxiety and depression have been reported in about 20% of pediatric patients with ADHD [18]; among adults, the prevalence of these comorbidities is up to 50% [33]. The respective prevalence of ADHD, anxiety, and depression have also been increasing in the US over the years, particularly among youths [34, 35], highlighting the urgency in addressing these psychiatric conditions at earlier stages to prevent their subsequent negative consequences in wider populations.

Findings of this study may help raise awareness to various stakeholders on the magnitude of the excess burden imposed by psychiatric comorbidities on pediatric patients with ADHD. For instance, parents and teachers may pay more attention to the emotional well-being of children and adolescents with ADHD and provide them with needed support to ease their anxiety or depression; clinicians may be more vigilant in considering the impact of psychiatric comorbidities in their ADHD management plan; and research efforts may be directed to exploring medications effective for improving both ADHD and comorbid psychiatric symptoms. These and other strategies may help alleviate the substantial burden associated with psychiatric comorbidities imposed on pediatric patients with ADHD and on the healthcare system at large.

### Limitations

This study is subject to limitations inherent to retrospective databases using claims data, including potential data omissions, coding errors, and the presence of rule-out diagnosis (i.e., a record of an ADHD, an anxiety, or a depression diagnosis does not necessarily indicate that the patient has a true diagnosis). In addition, while cohorts were balanced based on observable characteristics, there may be residual confounding due to unobservable confounders; therefore, no causal inference can be made from this retrospective observational study. It should also be noted that the burden associated with anxiety and depression goes beyond HRU and healthcare costs, but this study was limited to information available in claims data; future research is warranted to explore other types of burden associated with comorbid anxiety and depression in ADHD. Finally, this study included only commercially insured patients and may not be representative of the pediatric population with ADHD in the US.

## Conclusion

Comorbid anxiety and depression were associated with significantly increased risk of HRU and higher healthcare costs among pediatric patients with ADHD. In particular, the presence of both comorbid conditions resulted in 3.5 times higher excess costs relative to ADHD alone. Increased awareness of such burden and strategies to co-manage ADHD and psychiatric comorbidities are warranted to help mitigate the burden borne by patients and the healthcare system.

### Electronic supplementary material

Below is the link to the electronic supplementary material.

Supplementary Material 1

## Data Availability

The data that support the findings of this study are available from IQVIA. Restrictions apply to the availability of these data, which were used under license for this study. Data are available from the authors with the permission of IQVIA.

## Abbreviations

AMCP: Academy of Managed Care Pharmacy
FDA: Food and Drug Administration
HCUP: Healthcare Cost and Utilization Project
HIPAA: Health Insurance Portability and Accountability Act
HRSA: Health Resources and Services Administration
HRU: Healthcare resource utilization
NSCH: National Survey of Children’s Health
PPPY: Per-patient-per-year
SD: Standard deviation
US: United States
USD: United States dollar
