# Supplementary material for: Healthcare resource utilization and costs associated with psychiatric comorbidities in pediatric patients with attention-deficit/hyperactivity disorder: a claims-based case-cohort study
Source: Child Adolesc Psychiatry Ment Health. 2024 Jul 8;18:80. doi: 10.1186/s13034-024-00770-8 (PMC11232137; doi:10.1186/s13034-024-00770-8)
Supplement: Supplementary file 1 — Supplementary Material 1 [file 13034_2024_770_MOESM1_ESM.docx]

# SUPPLEMENTARY MATERIALS

## Supplementary Figure S1. Sample selection flowchart


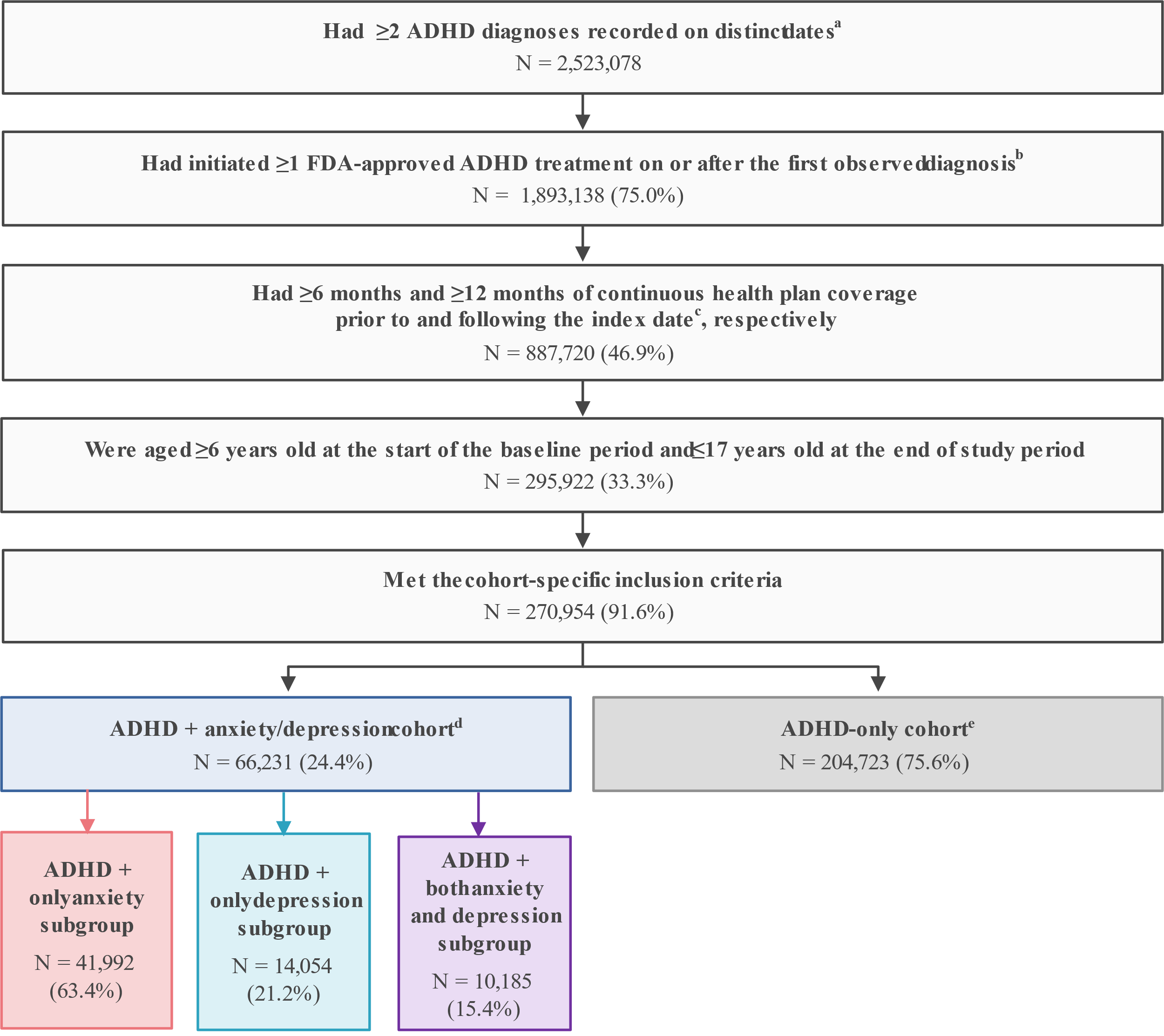


ADHD, attention-deficit/hyperactivity disorder; DSM-5, Diagnostic and Statistical Manual of Mental Disorders, Fifth Edition; FDA, Food and Drug Administration; ICD-10-CM, International Classification of Diseases, Tenth Revision, Clinical Modification.

**Notes:**

^a^ ADHD was defined as ICD-10-CM codes: F90.x.

^b^ Patients could have initiated more than one treatment (i.e., multiple candidate index dates).

^c^ The index date was defined as a randomly selected date on which an FDA-approved agent for the treatment of ADHD was newly initiated.

^d^ Patients had ≥1 diagnosis for anxiety, depression, or both, as defined by the DSM-5 recorded on a medical claim during the baseline period and ≥1 during the study period.

^e^ Patients had no diagnoses for anxiety nor depression recorded on a medical claim at any time during the baseline or study period.
